# Rebooting the human mitochondrial phylogeny: an automated and scalable methodology with expert knowledge

**DOI:** 10.1186/1471-2105-12-174

**Published:** 2011-05-19

**Authors:** Roberto Blanco, Elvira Mayordomo, Julio Montoya, Eduardo Ruiz-Pesini

**Affiliations:** 1 Departamento de Informática e Ingeniería de Sistemas, Universidad de Zaragoza, María de Luna 1, 50018 Zaragoza, Spain; 2 Instituto de Investigación en Ingeniería de Aragón, Universidad de Zaragoza, María de Luna 1, 50018 Zaragoza, Spain; 3 Departamento de Bioquímica y Biología Molecular y Celular, Universidad de Zaragoza, Miguel Servet 177, 50013 Zaragoza, Spain; 4 Centro de Investigación Biomédica en Red de Enfermedades Raras, Miguel Servet 177, 50013 Zaragoza, Spain; 5 Agencia Aragonesa para la Investigación y el Desarrollo, Miguel Servet 177, 50013 Zaragoza, Spain

## Abstract

**Background:**

Mitochondrial DNA is an ideal source of information to conduct evolutionary and phylogenetic studies due to its extraordinary properties and abundance. Many insights can be gained from these, including but not limited to screening genetic variation to identify potentially deleterious mutations. However, such advances require efficient solutions to very difficult computational problems, a need that is hampered by the very plenty of data that confers strength to the analysis.

**Results:**

We develop a systematic, automated methodology to overcome these difficulties, building from readily available, public sequence databases to high-quality alignments and phylogenetic trees. Within each stage in an autonomous workflow, outputs are carefully evaluated and outlier detection rules defined to integrate expert knowledge and automated curation, hence avoiding the manual bottleneck found in past approaches to the problem. Using these techniques, we have performed exhaustive updates to the human mitochondrial phylogeny, illustrating the power and computational scalability of our approach, and we have conducted some initial analyses on the resulting phylogenies.

**Conclusions:**

The problem at hand demands careful definition of inputs and adequate algorithmic treatment for its solutions to be realistic and useful. It is possible to define formal rules to address the former requirement by refining inputs directly and through their combination as outputs, and the latter are also of help to ascertain the performance of chosen algorithms. Rules can exploit known or inferred properties of datasets to simplify inputs through partitioning, therefore cutting computational costs and affording work on rapidly growing, otherwise intractable datasets. Although expert guidance may be necessary to assist the learning process, low-risk results can be fully automated and have proved themselves convenient and valuable.

## Background

Mitochondria are remarkable among eukaryotic organelles for possessing their own genome, which is inherited independently from the nucleus. Even though mitochondrial DNA (mtDNA) constitutes a very small fraction of the whole genome in higher organisms, the few genes it encodes are essential to cellular metabolism, and therefore main actors in the development of genetic diseases. Their functional roles grant them conservation and homogeneity; in fact, the analysis of their genetic variation is one of the primary sources of information for the inference of evolutionary relations in phylogenetics research.

In addition, mitochondrial DNA is an excellent intraspecies phylogenetic marker due to its strictly uniparental inheritance in most species and elevated mutation rate, and the structuring of its variability in humans indeed sheds light on many important questions [1]. To name a few, mtDNA-based phylogenetic studies have provided strong support for the African origin of the human species [2]; contributed to define the role of selection in human mitochondrial evolution [3]; and associated distinct mitochondrial genetic backgrounds to particular disease phenotypes [4], among others.

More specifically, this study relates to and shares some of the goals of the Human Genome Diversity Project (HGDP) [5], a worldwide survey of the genetic wealth of the human species undertaken shortly after the launch of the Human Genome Project. The HGDP ultimately aims to gain an understanding of genetic diversity patterns, their generative processes and evolutionary history. This, in turn, is expected to generate an immense amount of valuable biomedical information. Phylogenetics is naturally suited to the organization and analysis of genetic and variational data to elucidate the course of evolution that culminates in the observed diversity of the human species, as follows from the aims of the HGDP.

In this light, we set out to explore the systematic, escalated reconstruction of human mitochondrial phylogenies, gaining insight from present accomplishments and improving on the available methods and data to understand the evolution of mtDNA.

### Related efforts

The reconstruction of comprehensive phylogenies based on mitochondrial DNA is not a novel undertaking: the defining features of the molecule indeed make it an ideal target for such evolutionary studies. The number of available sequences has grown exponentially since the advent of frequent sequencing and submission of human mtDNA genomes to GenBank a decade ago. In particular, the number of published human mtDNA sequences has doubled over the last three years from approximately 4000 to more than 8000. Though certainly beneficial, the very fast pace of progress can undermine efforts to keep explanatory phylogenies up to date in detail. This steady growth represents an outstanding opportunity for comprehensive work on huge datasets and an excellent benchmark for all sorts of algorithms and techniques.

In this section we evaluate the merits and shortcomings of current alternatives and derive some convenient properties that will be incorporated into our proposal.

#### MITOMAP

MITOMAP [6] hosts what can be considered to this day the most comprehensive fine-grain human mitochondrial phylogeny available for general purpose. It was built from approximately 1000 mtDNA sequences and subsequently updated and maintained manually by Dr Wallace's group until the start of the ZARAMIT project [7] in 2007. The in-depth update process that it required is reportedly unfeasible as it cannot keep up with the growing number of published sequences; thus, it has not been updated since shortly before reaching the 3000-sequence milestone. Moreover, manual augmentation invalidates the formal principles of most construction methods: a mathematical optimality criterion and statistical support measures that lend credibility to a given tree.

Strictly speaking, the MITOMAP phylogeny is not a tree due to the introduction of reticulation events in those places where a simple, unequivocal path to each leaf cannot be determined (so some nodes may have two or more postulated parents, in contradiction with the mitochondrial mode of inheritance). Moreover, input sequences can be found associated to internal nodes instead of confined to leaf nodes, thus blurring the distinction between known hereditary relations and inferred ancestral sequences, commonly assumed to span all internal nodes. These irregularities encumber work with the tree and its evaluation, which is further encumbered by the lack of a machine-readable version of the phylogeny.

Lastly, the tree is enriched with abundant annotations on provenance, mutations and pointers to related literature. These important additions are largely dependent on supervised selection of features; consequently, actions should be taken to ensure coherence, avoid redundancy and decouple these steps from explicit tree upgrade operations.

#### PhyloTree.org

PhyloTree [8] is another recently started project that publishes periodically updated trees. Its focus is on haplogroup-like classification and as such offers very clean and simple results. It is a very useful resource, though several factors make it unsuitable for our purposes. First and foremost, it is a "tree skeleton" rather than a complete phylogeny and as such it does not offer a complete mutational landscape as a basis for in-depth genetic study. Secondly, like MITOMAP, it is, to a lesser extent, annotation-oriented, relying on curated bibliography listings, thus introducing a supervision factor that may prevent thoroughness to a degree. And thirdly, the details of the construction of the tree are unknown, as are the criteria for definition of new haplogroups and suppression of common polymorphisms. Nonetheless, as a repository of expert knowledge it can serve as an excellent guide for automated construction, as will be noted in the discussion of partitioning techniques for phylogenetic analysis below.

#### Other trees

Some recent studies such as [9] make use of special-purpose phylogenies which, though limited in their scope and extent, provide insight into desirable properties of the resulting trees, as well as common operations on them. These works could greatly benefit from the availability of high-quality, general-purpose phylogenies and in turn provide feedback to these. Lacking those, studies have to be conducted from scratch, most likely not aiming for automation, scalability or continuity.

Overall, there exist burdensome trends towards manual supervision or annotation, as well as a serious lack of information regarding construction methods and statistical support measures that could both be used to evaluate and improve existing trees. It is also apparent that expert knowledge plays an important role, which should be formalized and automated. Here we propose a methodology to automatically and efficiently build periodically updated phylogenetic trees where human supervision is kept to a minimum and does not disturb the reconstruction process itself.

## Results and discussion

### Sequences

Human mitochondrial DNA sequences are the raw materials of our study, processed all the way from public databases to annotated phylogenies. We consider both *flexible *and *strict *databases. The former encompass all available full sequences as defined by a suitable query, while the latter satisfy some additional quality constraints, the most significant being the suppression of sequences whose non-coding control region is unavailable.

The quality of strict sequence databases is especially relevant due to the conceptually simpler treatment that clean, uniform data allow. The effects of outliers and other anomalies should be mostly local, but incomplete sequences (in the form of gaps in the alignment) can severely limit the effective range of useful positions and decrease the resolution of the dataset. Although a loose database query can ideally avoid false negatives and ensure completeness, more extensive cleaning is needed to correct for the potentially larger number of false positives.

#### Sequence length tests

Almost all of the complete human mtDNA sequences have lengths in the 16550:16600 base pair range. No sequences can be found beyond the 16600 bp threshold. Sequences devoid of control region are clearly identified by the vacuum in the 15600:16300 range, supposing these are the only coherent "almost complete" sequences we allow (see *Sequence composition tests *below).

Outliers deserve special attention and, though automatically locatable, may require manual inspection prior to their inclusion in a dataset. All sequences in the 16500:16550 range belong to cancer tissue samples from [10] except for a healthy sequence with a 50-bp deletion [11]. The 16400:16500 range illustrates a rare 154-bp non-deleterious deletion [12] and thus is a legitimate sequence. Finally, two Indian sequences are found in the 16300:16400 range that appear to lack the first ~250 positions, though there is no mention to this irregularity in the original study [13] and the rest of its published sequences seem normal. For the sake of completeness and evaluation, none of these have been excluded from the study.

#### Sequence composition tests

Length criteria and formal sequence properties combined can yield a good approximation of the desired clean dataset, but data quality and query (or database) limitations must be taken into account to detect further false positives and disruptive data, including incomplete sequences that pass the simple length tests we have just described. Gaps neither have meaning nor are expected in raw sequences, though there is at least one case where the correction of a sequencing error (in the original human mtDNA reference sequence, predecessor to the current rCRS) has inserted an artificial *N *pseudodeletion to preserve the canonical numbering of positions established by its first, though faulty, incarnation. There is no simple way to tell apart these violations to the IUPAC nomenclature, which should be avoided using an additional "false gap" symbol, if possible.

**Figure 1:**
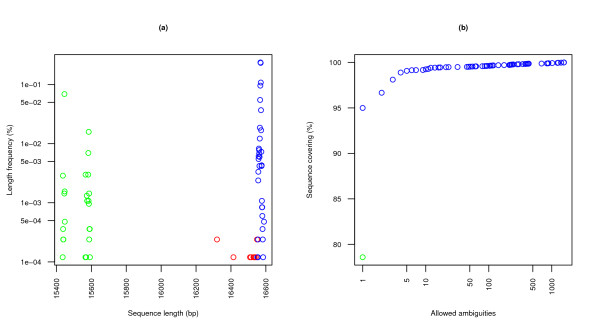
**Individual sequence features**. These descriptors operate directly on sequences. Due to their simplicity, they comprise the first tests to be applied to any prospective members of a dataset. **(a) **The *sequence length histogram *locates unusually short or long sequences, commonly classifying correct genomes as belonging to strict or flexible sets, and also detecting outliers which cannot be straightforwardly ascribed to either group. Blue dots mark accepted strict sequences; red dots, outlier strict sequences; and green dots, flexible and not strict sequences. **(b) **The *ambiguity covering histogram *serves as an aid for determining acceptable ambiguity thresholds and approximates a simple measure of aggregated quality. (The green dot shows the base covering of fully defined sequences, with zero ambiguous positions.)

Uncertainty in sequences (reflected in ambiguous or unknown positions) is undesirable because it blurs results and complicates their interpretation. These problems are widespread: from a strict database of 7395 full sequences, ambiguities exist in 24.0% of these, at least at the base pair level. We may accommodate an acceptable level of uncertainty by defining a maximum allowed number (or fraction) of ambiguous positions per sequence, hence adjusting the trade-off. We observe that a static threshold of 1 covers 95% of all sequences, whereas a threshold of 5 covers 99%. Sequences that exceed these boundaries significantly are clear candidates for inspection. Figure 1b shows database covering as a function of the ambiguity threshold. Flexible databases include a special case of partial sequences whose unknown control region can be considered a form of ambiguity, in the guise of long gaps at both ends of the alignment with no biological meaning. Its impact will be discussed in following sections.

#### Sequence identity

In large collections of densely sampled, closely related genomes, which are furthermore highly conserved and comparatively short in our case, not every sequence can be assumed to be unique as might be in other circumstances. Equality between two sequences does not provide any additional information to the single-sequence case, and this fact may be used to reduce effective datasets to some extent.

Within strict databases, 10.1% of all sequences may be thus compressed due to equality with others, which may act as their representatives until the final results are produced. Flexible databases include 944 additional sequences, 10.0% of which can be unified.

### Alignments

Construction of datasets for input to tree construction algorithms proceeds iteratively applying the procedures detailed in this section. Potential problems will be described together with the methods designed to solve them, as well as their effects on both inputs and outputs.

Each sequence is split prior to alignment according to the structural boundaries of coding and non-coding regions defined by the rCRS record on GenBank, resulting in 50 subalignments of lengths between 1-1812 bp (as defined by the reference) computed separately: 37 genes, 11 non-coding gaps in the coding region, and the D-loop, split in two by the numbering scheme. Their (overlapped) concatenation results in a full, 16832-position alignment where increases in sequence length are chiefly due to a handful of lineage-specific indels. Each partial alignment can be computed on a standard workstation in up to 6.5 hours for the biggest dataset and considerably less for medium-size inputs. Times increase somewhat for flexible databases, though total alignment costs remain comparable. Through this technique, otherwise problematic computations of large alignments become affordable.

Although pairwise alignments with the reference are generally required in order to robustly split sequences into their structural units (due to insufficient database annotation), these only need to be computed once and stored for future use, taking approximately 7 seconds each.

#### Simple results

Feeding an alignment algorithm with the results of a standard database query is a very straightforward procedure, but the results of this practice are rife with errors. Gene partitioning makes execution times manageable and to an extent structures the solution, but does not solve defects that are inherent to the sequences themselves.

The length of a direct alignment of the results of the MITOMAP query without any further preprocessing goes up to approximately 18000 characters. This is due to the fact that a few (*<*0.1%) complete sequences from [14,15] suffer from a bad definition of their starting position according to the reference: roughly speaking, the circular mtDNA chain has been cut at the wrong point for numbering purposes and canonical position 1 is found somewhere in the middle of the resulting string. These displacements produce an erroneous numbering in the affected sequences and, if left untreated, can severely damage alignment quality by creating very long gaps on both ends of the alignment, disrupting numbering and degrading otherwise usable characters in all other sequences. This, in turn, will have a negative effect on any phylogenies including such sequences.

#### Distance matrices and curation

Computation of pairwise distance matrices is not only a necessary step for some of the most popular tree building methods, but also a means of detecting uncommon sequences to check for false positives. Parsimony edit distances are a straightforward means of building a histogram and looking for outliers, though more sophisticated models could be used as well. We have found that all strict outliers within the accepted length ranges are actually incomplete sequences presenting long series of unknown positions, marked as *N*, belonging to just a few different studies [10,16-20]. Once these are treated, structural anomalies require more refined sequence checks.

**Figure 2:**
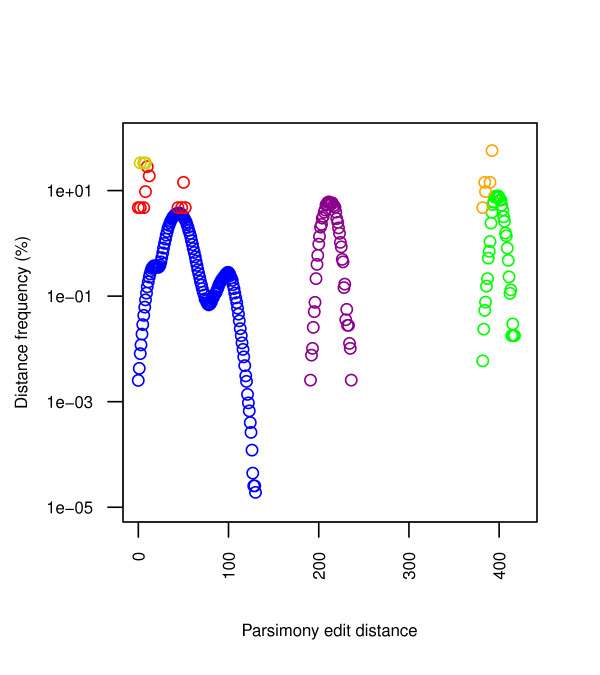
**Parsimony edit distances**. This figure plots the edit distance histogram of the strict database. The intraspecies markers are: blue dots for *H. sapiens *distances, red dots for *H. neanderthalensis*, and dark yellow dots for *H. sp. altai *(the latter two are clearly limited by the available sequences). Interspecies markers are: purple dots for *H. sapiens*-*H. neanderthalensis*, green dots for *H. sapiens*-*H. sp. altai*, and orange dots for *H. neanderthalensis*-*H. sp. altai*. The separation between all three species is clearly visible.

Once highly anomalous sequences have been removed, we are left with 7390 full sequences (6644 unique sequences), whose statistics are summarized in Figure 2. The distribution of *Homo sapiens *intraspecies distances, restricted for correctness to unambiguous sequences, follows a bimodal distribution with a main peak at 45 differing positions and a secondary peak at 100. Distances range between 0 and 130, with a combined average of 46.36 differences (*σ *= 16.26).

Predictably, the seven available *Homo neanderthalensis *sequences [21,22] and three *Homo sp. altai *sequences [23,24] are clearly separated from *Homo sapiens *sequences in terms of pairwise base differences. Generally speaking, whenever we consider clusters of statistically differentiated sequences (e.g., distinct species), these should be studied separately to avoid external sources of noise, determining relevant properties and outliers within each group; doing otherwise results in a mix of underlying distributions that sensibly complicates the problems of inference and detection. Sequences lacking their control regions could be studied jointly with full sequences if parsimony scores treat indels as single events; otherwise all sequences should be considered without their control region for comparability.

There is clearly some overlap with the simpler tests that have been described in preceding sections. The final battery of tests should be applied in order of ascending power (and complexity) to maximize efficiency. It is possible to compute edit distances from unaligned sequences, but a complete alignment is desirable for conducting more thorough examinations based on homology levels and conservation criteria, among others.

### Phylogenies

**Figure 3:**
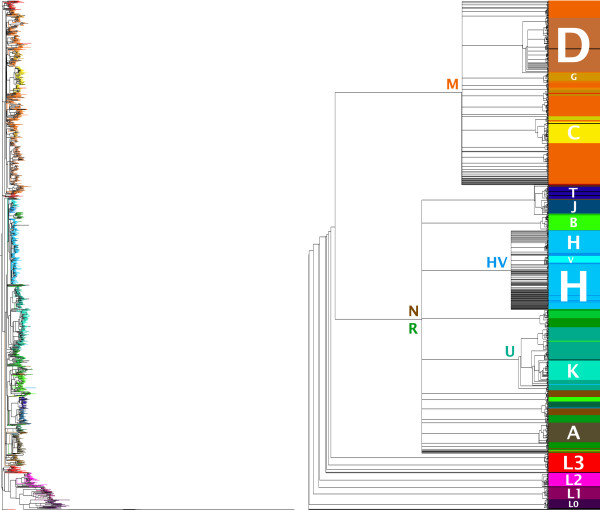
**Updated human mitochondrial phylogeny**. The left phylogram shows the base binary tree with its associated branch lengths (note the long Neanderthal and Altai clades next to L0). The right cladogram presents the aggregate bootstrap consensus and locates the main haplogroups as defined by MITOMAP's simplified lineages. Both trees are rooted according to the directionality of said classification.

Trees produced from curated alignments, shown in Figure 3, exhibit generally good properties. If we take MITOMAP's simplified mtDNA lineages [25] as the basis for a haplogroup classification and plot these groups on the resulting phylogenies, we observe that these qualitative properties and the relations between them are appropriately respected.

What local inaccuracies occur concern the exact situation of small haplogroups (i.e., those with a small number of specimens) or scattering of portions thereof in the tree, due to low relative weight and other phenomena. Although visually puzzling, it is correct to find some important parent haplogroups embedding their child haplogroups (and consequently "broken" into several parts) instead of indivisibly grouped together. This is reasonable because a child haplogroup is simply a convenient designation for a subtree within its parent; different subtrees need not be evolutionary siblings, though a detailed classification can certainly be beneficial. For purposes of visualization, emphasis should be placed on clearly marking parent clades as such.

If one is willing to sacrifice a small fraction of autonomy in the tree stage to guide the reconstruction process, it is possible to decree that a certain hierarchy of haplogroups (or clades, generally speaking) be imposed on any acceptable solution. In this case, the desired haplogroup hierarchy needs to be provided together with decision rules to classify individual sequences as pertaining to one particular group. A haplogroup subtree can be constructed for each group and later grafted into their combined supertree according to the postulated hierarchy, as we have described in [26]. As an added benefit, this strategy offers a great improvement in performance, and so it becomes feasible to employ sophisticated and comparatively costly algorithms.

The effect of bootstrap sampling on the trees is a trade-off between the inferred robustness of the reconstruction and the amount of potential blurring it may cause. Support tends to be very high near the leaves and decreases as we move higher up the tree. Polytomies result in those regions where a clearly dominant relation cannot be identified. This is particularly visible in the N haplogroup catch-all branch, which is the main point of dispersion in bootstrapped consensus trees. However, and although this represents a departure from the binary tree model, this compaction is coherent with established multifurcations at the haplogroup level.

#### Simple results

Flexible phylogenies feature very long gaps associated with missing information, even if curated databases are used. While these lacks are not enough to disrupt classification on a broad scale, they can entail a significant loss of fine grain resolution. Non-canonical start/end points have a similar effect on the alignment, inserting rows of gaps at both ends, as noted above.

All in all, artificial gaps degrade the effective performance of most tree reconstruction methods. The inclusion of these positions in the final labeled phylogenies is troublesome as these defects will be passed down to a large number of ancestral sequences, which will be plagued by many false indel events, completely unrelated to evolution. Therefore, it is advisable to either distinguish legitimate gaps from unsequenced regions, or else discard the latter altogether.

#### Quantitative analysis

**Figure 4:**
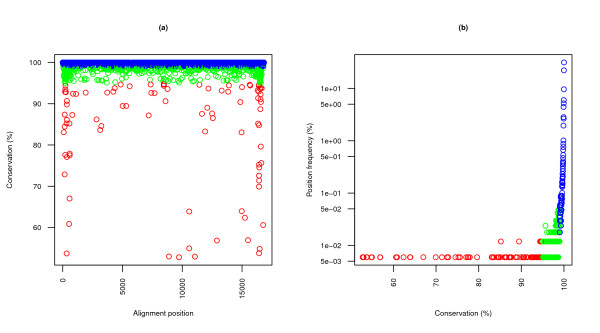
**Genetic conservation measures**. Conservation statistics are useful to evaluate single sequences and extract knowledge from the combined pool of available data. Incomplete sequences have to be discarded at least partially for results to reflect true polymorphic variations exclusively; the following plots do not include non-strict data. **(a) **The sequential conservation profile of the alignment indicates regions and positions of special interest. **(b) **When this profile is transformed into the *conservation frequency histogram*, some global trends become apparent. Blue dots are used for *α *≥ 0.99, green dots for 0.95 ≤ *α <*0.99, and red dots for *α <*0.95. Note that under these thresholds, the great majority of mutations affects conserved positions: *P*(*α <*0.95) = 0.570%, *P*(*α <*0.99) = 2.905%. In view of these extreme levels of conservation, it may be interesting to tune *α *to adjust the significance of "high" conservation levels to the raw amounts of closely related data.

The standard consensus (multifurcating) tree derived from the strict database of previous sections requires 67900 point mutations as defined by Fitch's parsimony algorithm (which, treating each character independently, does not unify indels). 31.21% of these involve at least one ambiguous position. 69.88% of all mutations affect highly conserved positions (*α >*0.95); this is expected due to the very high conservation of the alignment (*μ *= 99.80%, *σ *= 1.60%; see Figure 4): only 0.57% of all aligned characters fall below the 95% bound. Back-mutations occur in 24.91% of all point mutational events.

Table 1 summarizes the relations between the different types of basic events. Two interesting remarks arise from the data: first, ambiguous mutations are one of the main sources of disruption of conserved positions and never involve back-mutations (so their effects appear as local and close to the leaves); and second, many back-mutations are related to indels, suggesting a suboptimal treatment of these mutations. In fact, alignment columns with any number of gaps are usually ignored altogether due to the inability of typical substitution models to account for anything but simple substitution events.

**Table 1:** Point mutation statistics for the reference strict phylogeny

*Mutation type*	Conserved	Back-mutation	Conserved B-M	Remaining	Total
Unambiguous	27489	16915	4672	6976	46708

Ungapped	22158	6825	3365	2159	27777

Ambiguous	19960	0	0	1232	21192

Total	47449	16915	4672	8208	67900

Mutations are classified and counted according to their qualitative properties. Row categories separate perfectly defined mutations from those that involve ambiguous positions of some sort; "ungapped" unambiguous substitutions (mutations that do not involve gaps, i.e., indels) are given a separate category as well (Total = Ambiguous + Unambiguous, Unambiguous ≥ Ungapped). Columns relate mutations with alignment conservation information (95% threshold) and evolutionary history from the root of the tree to several types of significant events (Total = Conserved + Back-mutation - Conserved B-M + Remaining).

Changes are not uniformly distributed across clades. There is an average of 5 mutations per branch (*μ *= 4.68, *σ *= 34.66), though 20.1% of all branches are, in fact, empty. There may be up to 1574 events in a branch, but these are isolated cases that will be considered in the next section.

If all ambiguities were suppressed, a minimum of 7416 point mutations would be needed to build a perfect phylogeny [27] if at all possible, where for each character, every occurring symbol would have a single generation point (except the one found in the root of the tree). Therefore, the additional excess events indicate mutations that occur several times in the tree. As a matter of fact, some mutations are exceedingly common, arising up to 1256 times in the reference tree. However, all mutations with more than 100 generation points are either indels or transitions or involve ambiguous characters, and take place within the D-loop, except for positions 709 (transition), an insertion after 3105 (ambiguity) and 3107 (ambiguities and indels). The same trends are observed for mutations with 50-100 generation points, extended to a few other positions in a non-coding gap (8272-4 and 8281-3) and in coding regions: 3010, the insertion after 3105, 3106-7, 5460, 11914, 13708 and 15924.

**Figure 5:**
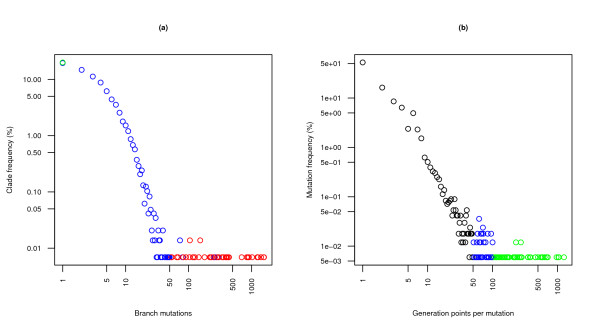
**Mutational statistics for the reference strict phylogeny**. Quantitative and statistical analysis of tree properties not only offers useful information about the evolutionary processes under study, but is also a very powerful means of detecting discordances at all stages of the reconstruction. Strict trees are particularly amenable to this treatment. **(a) **The histogram of point mutations per branch highlights typical patterns of evolution and identifies unusual and possibly error-prone generation points. Red dots represent outliers; and blue dots mark standard ranges and legitimate exceptions within the outlier range, with the green dot signaling the frequency of empty clades. **(b) **The histogram of individual mutation frequencies (i.e., the number of generation points for each mutation or group of related mutations) aims at the identification of especially important and recurrent mutations and the subsequent study of their patterns of generation.

Predictably, as we approach the common case of single point generation, more interesting mutations can be observed. Overall, there is a negative correlation between number of generation points and frequency, as seen in Figure 5b. The distribution is essentially the same whether or not we consider mutation variants unified by alignment position and/or include ambiguous mutations. We find 8328 fully defined mutations folded into 6328 positions, and 16991 total mutation types affecting 11520 positions, pointing to a significant decrease in (full) conservation levels exclusively due to sequence ambiguity.

#### Trees and dataset quality

A labeled phylogeny has the advantage of allowing sophisticated hypothesis testing. An obvious application of the inferred history of the tree is detection of clearly discordant sequences from which we may suspect an intrusion or defect of some sort.

In particular, an abnormally high number of branch mutations points to exceptionally divergent sequences, which should as such be inspected. Within our reference strict database and phylogeny, any node with more than 50 point mutation events is found to be a leaf associated with one of the studies referenced in *Distance matrices and curation *[10,16-20], except for the deletions reported in [11,12] and internal nodes joining the short sequences in [13] and the three following clades: *H. sp. altai *and *H. sapiens*-*H. neanderthalensis*, and *H. sapiens *and *H. neanderthalensis *each with the common ancestor of both. Below 50 events per branch we find legitimate changes, so more refined tests should be used to discern exceptional situations. Figure 5a shows both trends, which clearly follow different patterns.

Thus, the outliers that we found in sequences and alignments so far are reflected in the resulting tree if left untreated, and can be detected using tree-specific methods, though these require a higher overall cost due to their reliance on an existing phylogeny. Their impact within the tree appears as completely local and does not affect other regions of the tree; therefore, inclusion should be balanced against the marginally higher information these data may offer.

The suppression of affected terminal branches has, however, a significant effect on tree statistics; in particular, the number of point mutation events drops by 27.92% and most ambiguous mutations disappear. Thus, the tree approaches full definition, down to 2.91 mutations per character (from 4.03 in the initial tree), or 6.62 mutations per leaf (from 9.19). Note the significant decrease from random pairwise distances, indicating high compatibility.

Lack of information, as present in flexible phylogenies, has roughly the same effect as ambiguity, due to its equivalent representation within sequence alignments.

## Conclusions

### Sequences

We have automatically collected and sanitized all publicly available human mtDNA sequences, classifying them according to their completeness into flexible and strict databases. The former include all reasonably coherent sequences, a relatively heterogeneous set due to a historical pool of genomes whose control region is unavailable. The latter are restricted to structurally comparable, full sequences. Some preliminary tests (sequence length, composition, equality) on single sequences have further allowed us to cluster potentially related groups and isolate unusual data for inspection.

Many of the potential problems that need to be addressed arise from data ambiguity. Incomplete information is the most serious drawback, be it in the form of incomplete sequences or, especially, completely unknown positions which should be left for detection to subsequent steps. These imperfections blur to an extent the clean results that high-quality datasets should offer, as well as the simplicity of the methods upon which they rely.

In the future, the importance of correct representation of individual sequences and their underlying semantics should be stressed: the adoption of a formal ontology to describe sequences and their features would be of great aid for data classification and manipulation; it would also be of help to design simpler, more accurate queries. We will study integration and coherence between multiple primary data sources, as well, and application of sequence identity criteria to this end (we have addressed the latter in connection with parsimony models in [28]). Of special concern is the treatment of ambiguous characters according to their significance (be it missing information, artificial gaps or, most remarkably, heteroplasmy) and the adequate machine representation of sequences.

### Alignments

From partially curated sequence datasets, we have built high-quality alignments efficiently using structural subproblem decomposition techniques. We have subsequently used the results to study the relations between individual sequences and detect compositional anomalies by means of distance measures. The set of subproblems we have presented allows semantically sound, fast divisions which result in biologically meaningful subalignments. Whereas this basic partition suffices presently, we ought to consider the relative scaling of computing power and dataset growth (and associated processing costs). Should it become necessary to achieve further reductions in the overall cost of the alignment, unambiguous, conserved regions could be used to perform intragene splitting. The number of sequences per individual alignment could also be reduced by classifying and clustering related sequence groups.

Simple edit distances have been used to perform basic data classification related to automated curation processes. The intraspecies and interspecies group distributions reported in [21,23] have been confirmed and refined with extensive *Homo sapiens *mitochondrial datasets. These results encourage us to research improved preprocessing and clustering measures; distances can be computed using special-purpose, possibly exact, pairwise alignment algorithms such as Needleman-Wunsch and Smith-Waterman [29,30]. Legitimate yet incomplete sequences (i.e., those found in flexible sets and absent from strict sets) may be processed separately to guarantee homogeneity, or jointly with homologous regions of complete sequences as well, depending on distance models. Likewise, the effects of ambiguity in sequence alignment should be investigated more thoroughly.

Although we have removed especially disruptive data from our input sets, some conditions --displaced position numbering in particular-- may be corrected automatically. This, however, requires that local databases store corrected sequences, overriding any bad copies found in public databases until an update is made, at which point a renewed quality check could be made. Such procedures preclude treatment of mtDNA sequences as circular in favor of simpler, conventional methods. Moreover, we intend to exploit the structure and conservation of the human mtDNA molecule to achieve further improvements in computational costs and alignment quality.

### Phylogenies

We have demonstrated the applicability of our approach reconstructing updated, current and complete human mitochondrial phylogenies integrating the control region in the analysis; and carried out some preliminary analyses on them, using the trees to test several quality assessment criteria as well. The main improvements over previous phylogenies are: the use of a well-founded, systematic methodology, which spans all stages of the reconstruction; the exposition of said methodology; the study of its scalability and repeatability over time and growing datasets; and the customizability of the procedures according to the requirements of both inputs and outputs.

Efficiency has been achieved by means of a combination of biologically sound problem partitioning and effective parallelization of compatible subproblems through distributed systems. Thus, algorithm complexity is offset and problem complexity turned into computational advantage: periodic reconstruction becomes feasible, as does accommodation of dataset growth. To this end, both fundamental problem dimensions (number of sequences and sequence length) can be attacked through known or inferred properties.

As a result we produce automated (save for inclusion of dubious data), high-quality trees which, coupled with an appropriate computational framework, yield workable representations, which we can annotate, extend and analyze easily, as we have done to produce some of the results presented throughout the paper. Some interesting problems remain to be dealt with in the near future. From the end user standpoint, the ability to define and add attributes to the tree, as well as query and interact with it, is fundamental (we have recently addressed this problem in [31]). The main shortcomings concern visual interaction with such huge trees, particularly in combination with annotations and intensive exploration of these. On the other hand, most formats lack extension capabilities; we have found phyloXML [32] to be the only reasonable choice for such complex tasks.

Besides user-defined custom rules, special-purpose attributes and filters could be defined to analyze biological patterns of sequence quality and mark leaves as potential outliers, if not removed in previous sanitizing steps; likewise, such procedures could be applied iteratively to refine the original datasets. Another obvious improvement is the elaboration (and automation) of an adequate descriptive formalism for mutations: for instance, merging indels; detecting, if applicable, the gene where the mutation takes place, whether it is synonymous or else what change it effects on the amino acid sequence.

Yet another interesting aspect concerns qualitative evaluation and comparison between different alignments and trees. This comprises everything from model selection [33] and sensitivity analysis to posterior tree scoring and topological distances. A related prospect deserving of further attention is the addition of general constraints to reflect known biological properties, which may further simplify certain tasks and favor decomposition as usual, possibly including past results as guidelines. The conservation of such properties in the outputs can be used as a qualitative measure of correctness, as well.

In addition to phylogeny-supported curation, it is possible to conceive procedures for tree-driven data correction, determining the simplest ways to integrate discordant data in a way that is consistent with the scoring model. These ideas can be of use to resolve ambiguity and elegantly integrate lacking regions in flexible databases without greatly affecting tree scores, as is usually the case when unknown information is treated as absence of biological features.

Tree optimality and robustness are among the most difficult qualities to evaluate. Statistical methods provide an approximation to these problems, subject to a certain evolutionary model, at the cost of greatly increased computational loads. In addition, more general phylogenetic networks could be used to mark ambiguous hotspots while retaining the information of the main tree. Likewise, polytomous trees are not strictly undesirable, since consistently unresolved binary nodes may point to relevant evolutionary properties, as has been noted before.

To summarize, our intent is to keep improving tree reconstruction from both computational and biological standpoints, as much as to add and extract useful information from the results. We believe formalization of knowledge and automation are keys to carry out these objectives, supported by expert assistance to the information systems designed to this end. As phylogenies become recipients and organizers of information, interoperability with external systems becomes of the utmost importance.

Both biological and computational goals can be greatly aided by integration and cooperation with existing efforts in the study of human mitochondrial diversity at the sequence level, such as MITOMAP [6] and HmtDB [34], and at the tree level, like PhyloTree [8]; this should be one of the first steps to take. Additionally, improved and specialized algorithms can take advantage of the special structural features of mtDNA and the size and density of growing datasets, both to learn or infer new information from these and to use it to assist in and improve the reconstruction of phylogenies. Finally, reliable information systems must be matured to handle all these tasks and make them easily available to researchers.

### Availability

Updated phylogenies and research results are made available through the project website at [35]. Trees can be browsed online by means of the ATV-derived Archaeopteryx applets [36], and downloaded for local use. We plan to publish different versions of each tree, including enriched and cross-referenced phylogenies, related data and continuing research.

## Methods

**Figure 6:**
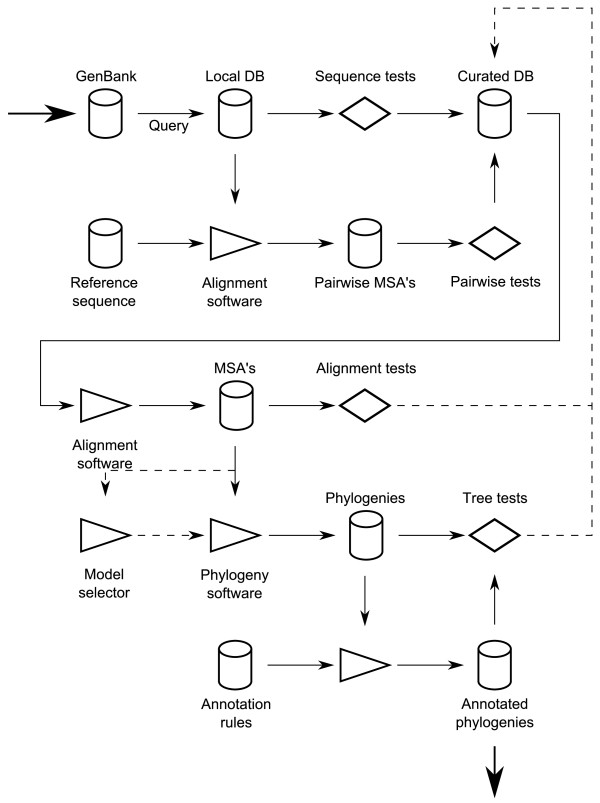
**System workflow architecture**. Individual algorithms and testing procedures are integrated into a workflow that directs and automates their interactions. Storage stages are interleaved with transformation stages, which are either algorithmic (marked as arrow-shaped triangles, they explicitly advance the resolution of the problem associated to their input) or restrictive (marked with a diamond shape, these tests refine algorithmic inputs diverting simple flows through feedback loops to previous storage stages). Note many transformation stages are actually concurrent scatter-gather processes (e.g., gene alignments, bootstrap replicates, etc.).

Each stage of the reconstruction requires specific algorithms (and software that implements them) and tests of increasing complexity and power, which are summarized under the following headings. These processes are arranged into a workflow (see Figure 6) that describes their constructive interactions and feedback loops, and ultimately documents and directs the execution of jobs.

### Sequences

The selection of input sets is a trade-off between simplicity and inclusiveness, and a demand for high-quality data; it should be kept in mind that treatment of gaps is suboptimal under practically any tree reconstruction method. The simplest preprocessing is the removal of ambiguous or incomplete sequences, and further sieves are supported by alignments and phylogenies.

GenBank [37] is the primary source of our data. Sequence identity, as discussed above, could be a simple method to incorporate additional sources while avoiding consistency problems, as well as to determine sequence exclusion. The current MITOMAP query on GenBank (*Homo[Organism] AND gene in mitochondrion[PROP] AND 14000:19000[SLEN] NOT pseudogene[All Fields]*) is used as a starting point, though caution must be exercised due to the partly unstructured nature of database submissions [38]. Query correctness and database curation are of paramount importance to subsequent steps of the reconstruction, locating and isolating suspicious entries for expert supervision and incorporating new findings as formal procedures and rules. False positives (i.e., intrusions) should be kept to a minimum or eliminated; false negatives (i.e., absences) are undesirable, though they do not harm accuracy. Outliers are detected by comparative and compositional tests of various types.

### Alignments

Alignment quality is also critical to the correctness of the results. Unlike sequence curation, sequence alignment is a very complicated algorithmic problem for which no efficient exact methods exist either in general or simplified models [39,40]. However, finding a (near-)optimal alignment of closely related sequences is significantly easier, since their conserved structure make good solutions easy to spot. Exploiting unambiguous biological units within sequences (e.g., genes), the problem is split in a set of smaller instances. Thresholds are inferred robustly by pairwise alignment with a known reference sequence (rCRS in human mtDNA [41,42]). It is necessary to contemplate overlaps between adjacent units and two types of indel events in protein-coding genes: full indels operating at the codon level and frameshift mutations. Finally, the results are merged into the complete alignment.

Furthermore, it is necessary to recognize defective alignments and identify the data that cause their degradation. Alignments allow complex quality tests, which we can classify according to their scope:

• Pairwise tests, usually against a reference sequence, which estimate the fitness of an individual datum.

• Global tests that detect statistical outliers and survey the underlying structure of the dataset.

It must be borne in mind that alignment algorithms may not respect any standard biological nomenclature. MUSCLE [43] has been used to produce the results presented in our study.

Alignments can be used to detect global problems, but also to study anomalies within individual sequences. A pairwise alignment of a sequence with a canonical reference is useful for conducting classification and other analyses. Edit distances computed against a high-quality reference can be used to detect potentially troublesome sequences, as well. These methods generalize some direct composition tests on individual sequences. Acceptable thresholds can be set to find anomalies in all these tests.

### Phylogenies

The phylogeny reconstruction problem is known to be NP-complete [44,45], and most approximate methods scale poorly with problem size. However, even for our target datasets, it is possible to employ maximum likelihood methods efficiently in a parallel execution environment and with some performance tweaks. Using jModelTest [46] and random sampling of the complete alignment, we have confirmed the GTR model with gamma-distributed rate heterogeneity among sites as the best fit for human mtDNA, even when penalized by information criteria; invariant sites have no observable effects. Robustness is assessed by fully parallel bootstrap sampling [47,48]. The *H. neanderthalensis *and *H. sp. altai *sequences pinpoint the root of the tree and no external outgroups are needed. RAxML [49] has been used as the software engine using the GTRCAT approximation to rate heterogeneity [50], with model parameters estimated for each bootstrap sample.

Evaluation of the resulting trees can obey to qualitative (e.g., verification of structural properties, population haplogroups) and quantitative criteria (e.g., bootstrap estimates). The fit of sequences and branches (subtrees) can be determined by defining relevant features (e.g., number of mutations per branch) and inspecting their outliers. Comparison between different candidate phylogenies, and possibly different datasets, requires a well-defined scoring framework, for which parsimony schemes offer useful metrics independent of problem dimensions. Average number of mutations per sequence (and position, if needed) and mutations per branch are two such measures.

Automated and extensible annotation is one of the defining features of our approach. The first step from a basic phylogeny to one that can be used to assist in sophisticated studies is the labeling of its branches with their hypothesized mutation events. The mutational history can be derived from the inferred ancestral sequences; for this we use generalized Fitch parsimony [51] due to its intuitive interpretation and implicit resolution of ambiguities. Enriched labels can include additional information determined by user-defined rules. We have applied custom rules to detect higher-level events that can be of interest to detect potentially deleterious and rare mutations. In particular, mutations affecting extremely conserved characters in the alignment (defined by a custom dominant fraction *α *≲ 1) may have important phenotypic effects and so should be monitored. Likewise, back-mutations, which reverse changes occurred during the evolution of the sequence from the root to the leaves, are marked for inspection. Other rules can be added to perform other thorough analyses and integrate their results into the tree.

## Authors' contributions

RB designed and implemented the system, performed the analyses and drafted the manuscript. EM designed the system and revised the manuscript. JM and ERP designed the experiments. All authors read and approved the final manuscript.
